# Phase Partitioning of GM1 and Its Bodipy-Labeled Analog Determine Their Different Binding to Cholera Toxin

**DOI:** 10.3389/fphys.2017.00252

**Published:** 2017-05-09

**Authors:** Sami Rissanen, Michal Grzybek, Adam Orłowski, Tomasz Róg, Oana Cramariuc, Ilya Levental, Christian Eggeling, Erdinc Sezgin, Ilpo Vattulainen

**Affiliations:** ^1^Department of Physics, Tampere University of TechnologyTampere, Finland; ^2^Paul Langerhans Institute Dresden of the Helmholtz Centre Munich at the University Clinic Carl Gustav Carus, TU DresdenDresden, Germany; ^3^German Center for Diabetes ResearchNeuherberg, Germany; ^4^Department of Physics and Energy, University of LimerickLimerick, Ireland; ^5^Department of Physics, University of HelsinkiHelsinki, Finland; ^6^Department of Integrative Biology and Pharmacology, University of Texas Health Science CenterHouston, TX, USA; ^7^MRC Human Immunology Unit, Weatherall Institute of Molecular Medicine, University of OxfordOxford, UK; ^8^MEMPHYS–Center for Biomembrane Physics, University of Southern DenmarkOdense, Denmark

**Keywords:** GM1, ganglioside, cholera toxin, membrane domains, molecular dynamics simulations, model membranes

## Abstract

Driven by interactions between lipids and proteins, biological membranes display lateral heterogeneity that manifests itself in a mosaic of liquid-ordered (Lo) or raft, and liquid-disordered (Ld) or non-raft domains with a wide range of different properties and compositions. In giant plasma membrane vesicles and giant unilamellar vesicles, specific binding of Cholera Toxin (CTxB) to GM1 glycolipids is a commonly used strategy to label raft domains or Lo membrane environments. However, these studies often use acyl-chain labeled bodipy-GM1 (bdGM1), whose headgroup accessibility and membrane order or phase partitioning may differ from those of GM1, rendering the interpretation of CTxB binding data quite problematic. To unravel the molecular basis of CTxB binding to GM1 and bdGM1, we explored the partitioning and the headgroup presentation of these gangliosides in the Lo and Ld phases using atomistic molecular dynamics simulations complemented by CTxB binding experiments. The conformation of both GM1 and bdGM1 was shown to be largely similar in the Lo and Ld phases. However, bdGM1 showed reduction in receptor availability when reconstituted into synthetic bilayer mixtures, highlighting that membrane phase partitioning of the gangliosides plays a considerable role in CTxB binding. Our results suggest that the CTxB binding is predominately modulated by the partitioning of the receptor to an appropriate membrane phase. Further, given that the Lo and Ld partitioning of bdGM1 differs from those of GM1, usage of bdGM1 for studying GM1 behavior in cells can lead to invalid interpretation of experimental data.

## Introduction

Glycosphingolipids (GSLs) are important constituents of cell membranes, participating in a wide range of biological processes such as recognition of hormones, function of bacterial and viral toxins, cell growth/differentiation, and cell-cell interaction (Karlsson, 1989; Miljan and Bremer, 2002; Ewers et al., 2009). The key to understanding GSL function is the conformational behavior of GSL headgroups. Molecular dynamics (MD) simulations and structural data for the GM1 headgroup-lectin interactions have suggested GSL headgroups to undergo differential conformational selection (Lingwood et al., 2011; Blaum et al., 2016). Interestingly, GSLs are often referred to as being “cryptic,” which stems from the observation that their recognition is regulated by the physicochemical properties of the proximal membrane environment, such as membrane fluidity influenced by, e.g., cholesterol concentration and protein content (Shichijo and Alving, 1985; Lampio et al., 1986; Stewart and Boggs, 1993; Kiarash et al., 1994; Mahfoud et al., 2010; Sezgin et al., 2012b). These findings suggest that the conformational landscape of the GSL headgroup is subject to various physical factors such as spatial and electrostatic effects imposed by the headgroup's chemical structure and its molecular interactions. Differential ligand recognition of discrete pools of GSLs in native membrane environments has also been suggested to play an important role in triggering specific signaling pathways (Haselhorst et al., 2001; Blaum et al., 2016). Therefore, it is important to understand the molecular mechanism of how membranes and their physicochemical properties modulate GSL headgroup presentation and availability.

**Figure 1:**
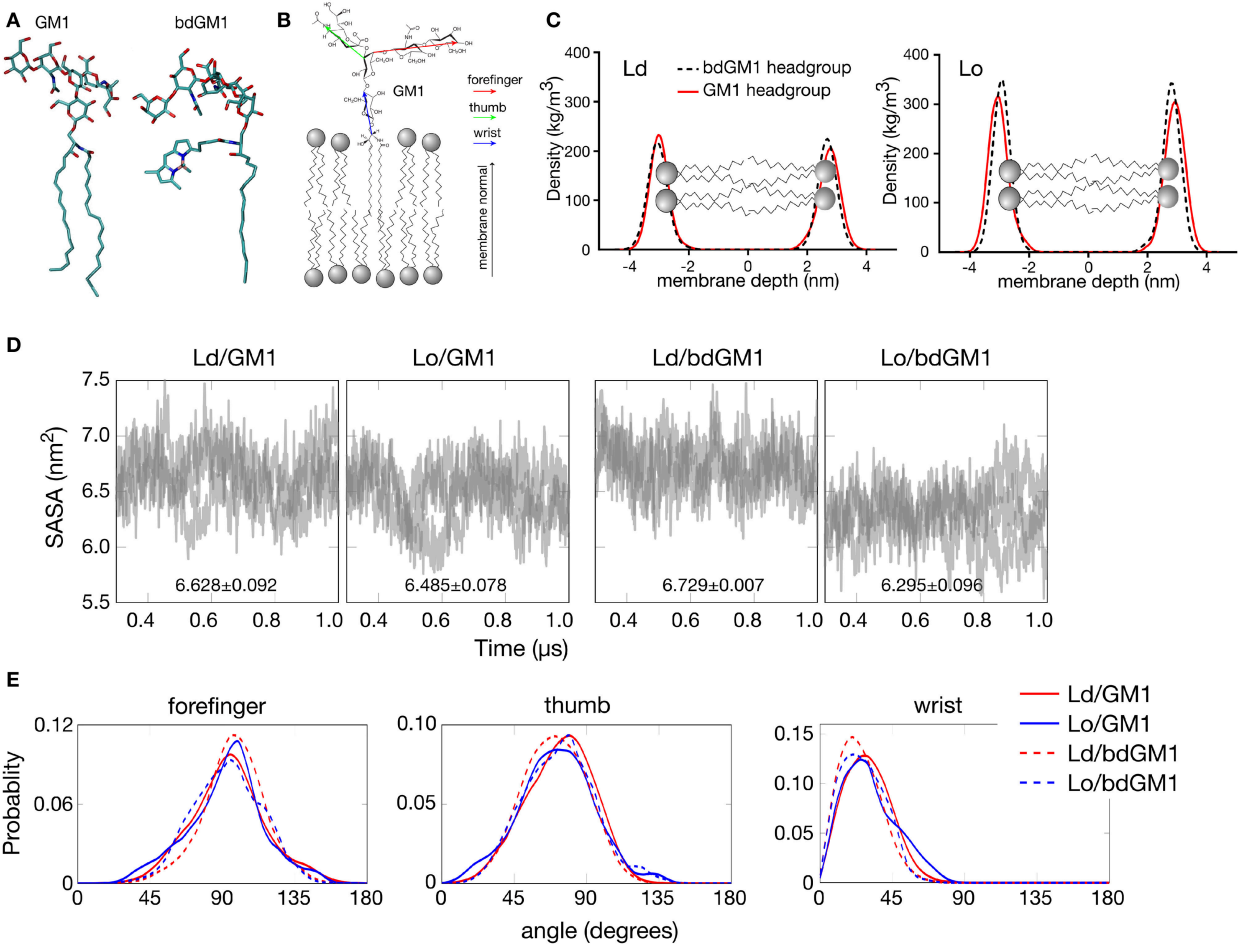
**Headgroup geometry of GM1 and bdGM1 in the Lo and Ld phases. (A)** Snapshot of GM1 and bdGM1 taken from the atomistic MD simulations. **(B)** Illustration of the vectors representing the different GM1 headgroup subunits. **(C)** Density profiles of atoms from GM1 headgroups in membranes that are in the Ld and Lo phases (in systems 5–8, Table S1 without CTxB). Here, a value of zero in membrane depth corresponds to the bilayer center. The differences in the absolute density values for the GM1 headgroups are attributed to the presence of cholesterol, which is significantly smaller than the other surrounding lipids, thus reducing the dimensions of the simulation box in the Lo system. **(D)** Results for the solvent accessible surface area (SASA) calculated per molecule for GM1 and bdGM1 headgroups (see also Figure S3). In each graph, the results of three replicas are shown by means of gray transparent lines. **(E)** Angle distributions in Ld and Lo for the different subunits of GM1 and bdGM1. The angle is defined with respect to the membrane normal, thus a value of zero corresponds to a situation where the vector stands upright along membrane normal, and an angle of 90° describes a vector lying along the membrane surface. **(D,E)** are based on data with CTxB, but the results have been computed from the leaflet not bound to CTxB.

Among the many GSLs, GM1 (Figure 1A) is used as a default lipid marker for the nanoscopic cholesterol/sphingomyelin (Chol/SM) enriched, liquid-ordered (Lo) membrane domains usually referred to as “membrane rafts,” opposing liquid-disordered (Ld) environments (Harder et al., 1998; Bacia et al., 2005; Ewers et al., 2009; Sezgin et al., 2012a,b). In cell membranes, cell-derived giant plasma membrane vesicles (GPMVs), and synthetic giant unilamellar vesicles (GUVs), GM1 detection by fluorescently labeled Cholera Toxin (CTxB) is a well-established tool for monitoring Lo membrane domains. Alternative and more direct reporters of GM1 are fluorescent analogs such as the acyl chain-bodipy labeled GM1 (bdGM1, Figure 1A). However, these GM1 analogs are not identical to native GM1 in terms of their biochemical and biophysical behavior, and it is not clear whether the conformational distributions of GM1 and bdGM1 headgroups are alike, as they should be if bdGM1 were used to consider CTxB binding with GM1. Further, bdGM1 is excluded from Lo domains in GUVs, while it is equally distributed in GPMVs (Sezgin et al., 2012a,b). Toxin binding, however, occurs in both systems exclusively in the Ld phase, which gives rise to a question of the importance of membrane phase in CTxB binding. Given the importance of GM1-CTxB binding in initiation of specific signaling pathways at cell surfaces, determination of the principles guiding the binding of CTxB to GM1 and bdGM1 is called for.

Here, we used extensive atomistic MD simulations supported with CTxB binding experiments to investigate how the binding of CTxB to its receptor (GM1, bdGM1) is modulated by the receptor's membrane phase partitioning. The results highlight the importance of ganglioside partitioning in CTxB binding and the great care needed in interpreting results based on the use of labeled ganglioside receptors.

## Results

### Atomistic simulations highlight subtle differences between GM1 and bdGM1 headgroup conformations

CTxB is a pentameric protein with five GM1 binding sites (Merritt et al., 1994). Therefore, geometrical compatibility between CTxB and GM1 molecules in the plane of a membrane is essential for efficient binding (Ewers et al., 2009). Given this, any structural modifications or GM1 reorganization in the membrane plane could compromise the binding. This would likely take place if a fluorophore were attached to GM1, which is the case with bdGM1, or if the state of liquid ordering of the surrounding lipid matrix would change, which is the case if CTxB binding would take place in Ld instead of Lo. To explore the consequences of these scenarios, we performed all-atom molecular dynamics (MD) simulations on GM1 and bdGM1 (Figure 1A) in both Lo (N-stearoyl-D-erythro-sphingosylphosphorylcholine/cholesterol (SSM/Chol)) and Ld (1,2-dioleoyl-sn-glycero-3-phosphocholine (DOPC)) membranes. Lo membranes were composed of ~46 mol% Chol, ~46 mol% SSM, and ~8 mol% GM1 (or bdGM1). Ld membranes were comprised of ~92 mol% DOPC with about 8 mol% GM1 (or bdGM1). Both Lo and Ld membranes were studied both with and without CTxB (see Table S1, see Supplementary Information (SI)). The key microsecond-simulations were carried out in three replicas. Details of the simulation models, simulations, and experimental methods and materials are discussed in SI.

First we analyzed how the GM1 headgroups are localized and oriented. We therefore calculated the mass density profiles of atoms from the GM1 headgroup and its components subdivided in thumb, forefinger, and wrist subunits (Figures 1B,C and Figures S1, S2). No significant differences between the Lo and Ld environments were observed in the localization of the GM1 headgroups for either GM1 or bdGM1, except for a minor shift toward the membrane core for bdGM1 in the Lo phase (Figure 1). This effect was strongest for the thumb and the forefinger rings of the bdGM1 headgroup (Figure S2). The results for the mass density profiles were confirmed by data for the solvent accessible surface area (SASA), which is the molecule's surface area that is accessible to solvent (in this case, water) (for the definition of SASA, see SI). SASA provides an estimate for the exposure of the GM1 headgroup to the water phase above the bilayer core and therefore indirectly indicates to what extent the headgroup is oriented toward the water phase. Only for bdGM1 a pronounced SASA change of ~0.43 nm^2^ per molecule between Ld and Lo was observed, indicating a higher exposure of the bdGM1 headgroup in Ld (Figure 1D and Table S2). The difference in SASA for bdGM1 was mainly caused by a change in the orientation of the thumb region (Figure S3). Overall, these observations may partially explain the preferential CTxB binding of bdGM1 in the Ld phase of phase separated model membranes (Sezgin et al., 2012b).

To quantitatively determine whether these differences stem from altered headgroup conformations, we calculated the angles between membrane normal and vectors of the respective GM1 headgroup components. Here, we found that for both GM1 and bdGM1 the conformation of the headgroup is very similar—the forefinger is tilted toward the membrane, while the thumb and the wrist subunits are more exposed to water (Figure 1E and Table S3)—largely regardless of the molecular structure and membrane environment. However, while the overall changes between the four systems are relatively small (Figure 1E and Table S3), there are a few interesting features that may be involved in the altered GM1-CTxB binding and thus need to be highlighted. First, the bodipy label orients the ganglioside wrist subunit toward the water phase by ~5°, and this result holds in both lipid environments when compared to unlabeled GM1. Second, if the bodipy marker is attached to GM1, the thumb part of the GM1 headgroup is tilted slightly (by ~3°) toward the water phase in Ld, but (by ~2°) toward the membrane in Lo, which largely explains the difference observed in the mass density profile in the Lo phase (Figure 1C). The forefinger subunit of bdGM1 is oriented slightly more toward the membrane than the forefinger of GM1. In the case of bdGM1, this orientation in favor of the membrane surface is larger in Ld than in Lo. In the case of GM1, the forefinger in Lo tilts to some extent more toward the membrane surface than in Ld. These differences are not substantial but anyhow evident. The majority of the change in the forefinger orientation is caused by the terminal sugar residue (β-galactose; Figure S4, and Table S4).

The results in Figure 1E suggest that the conformation of GM1 with respect to the membrane plane is not dependent on cholesterol. Meanwhile, recent MD simulations on POPC/cholesterol/GM1 membranes (with a ratio of 75/20/5) and experiments are in favor of the opposite view (Lingwood et al., 2011). We therefore explored this matter by considering the conformation of GM1 in a DOPC/cholesterol/GM1 membrane with ~46 mol% DOPC, ~46 mol% cholesterol, and about 8 mol% GM1, where the relative amounts of these lipid types match those used in the present Lo bilayers with SSM, cholesterol, and GM1. The results (Figure S5) show that in the DOPC/cholesterol/GM1 membrane the headgroup of GM1 is strongly tilted against the membrane, in agreement with ref. (Lingwood et al., 2011), the tilt angle of the GM1 headgroup in this case being much larger than in SSM/cholesterol/GM1 membranes. Clearly, the GM1 headgroup orients in a cholesterol-dependent manner, however the significance of cholesterol depends on the lipid pool hosting cholesterol.

**Figure 2:**
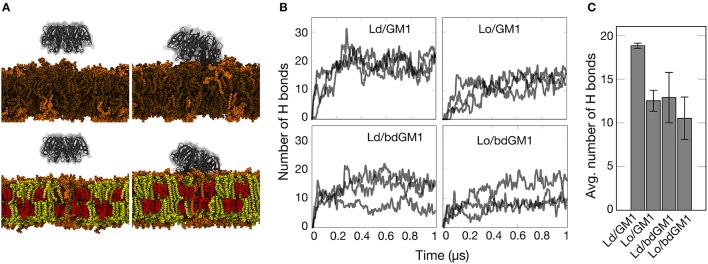
**Simulation results gauging the dependence of GM1-CTxB binding on membrane environment in simulations of systems with CTxB. (A)** Snapshots of simulated membrane systems at 0 (left) and 500 ns (right) in Ld (upper) and Lo (lower) phases (Lo composition: 46 mol% Chol, 46 mol% SSM, and 8 mol% GM1 (or bdGM1); Ld composition: 92 mol% DOPC and 8 mol% GM1 (or bdGM1)). DOPC is depicted in brown, cholesterol in red, SSM in yellow, and GM1 in orange. Water molecules are not shown for clarity. **(B)** Time course of the number of hydrogen bonds established between GM1 species and CTxB. Each case was simulated three times. **(C)** Average number of hydrogen bonds (H bonds) established between GM1 species and CTxB between 300 and 1,000 ns. The error bar represents the standard error.

The observed conformational differences prompted us to follow the availability of the headgroup for ligand binding. We therefore simulated the actual binding of CTxB to both GM1 and bdGM1 in Ld and Lo environments (snapshots taken from simulations shown in Figure 2A). As Figure 2B illustrates, the binding of CTxB to the membranes took place rapidly in about 20-100 ns. The number of hydrogen bonds established between the protein and the respective GM1 molecules was used as a correlate for the binding affinity (Figures 2B,C and Table S5). For GM1, this analysis revealed a greater number of CTxB-GM1 hydrogen bonds in the Ld phase, suggesting a slight preference for CTxB binding to the Ld phase (Figure 2C, and Tables S5). For bdGM1, the effect was similar but quite a bit weaker. These conclusions are supported by consideration of contacts between CTxB and GM1 (or bdGM1), (Table S6), using 0.35 nm as the maximum distance for a contact. This analysis revealed that the number of GM1/bdGM1 bound to CTxB was 8.9 ± 0.7 (GM1 in Ld), 6.8 ± 1.0 (GM1 in Lo), 5.6 ± 1.4 (bdGM1 in Ld), and 6.9 ± 0.9 (bdGM1 in Lo).

### Experiments point to differences in membrane phase partitioning

**Figure 3:**
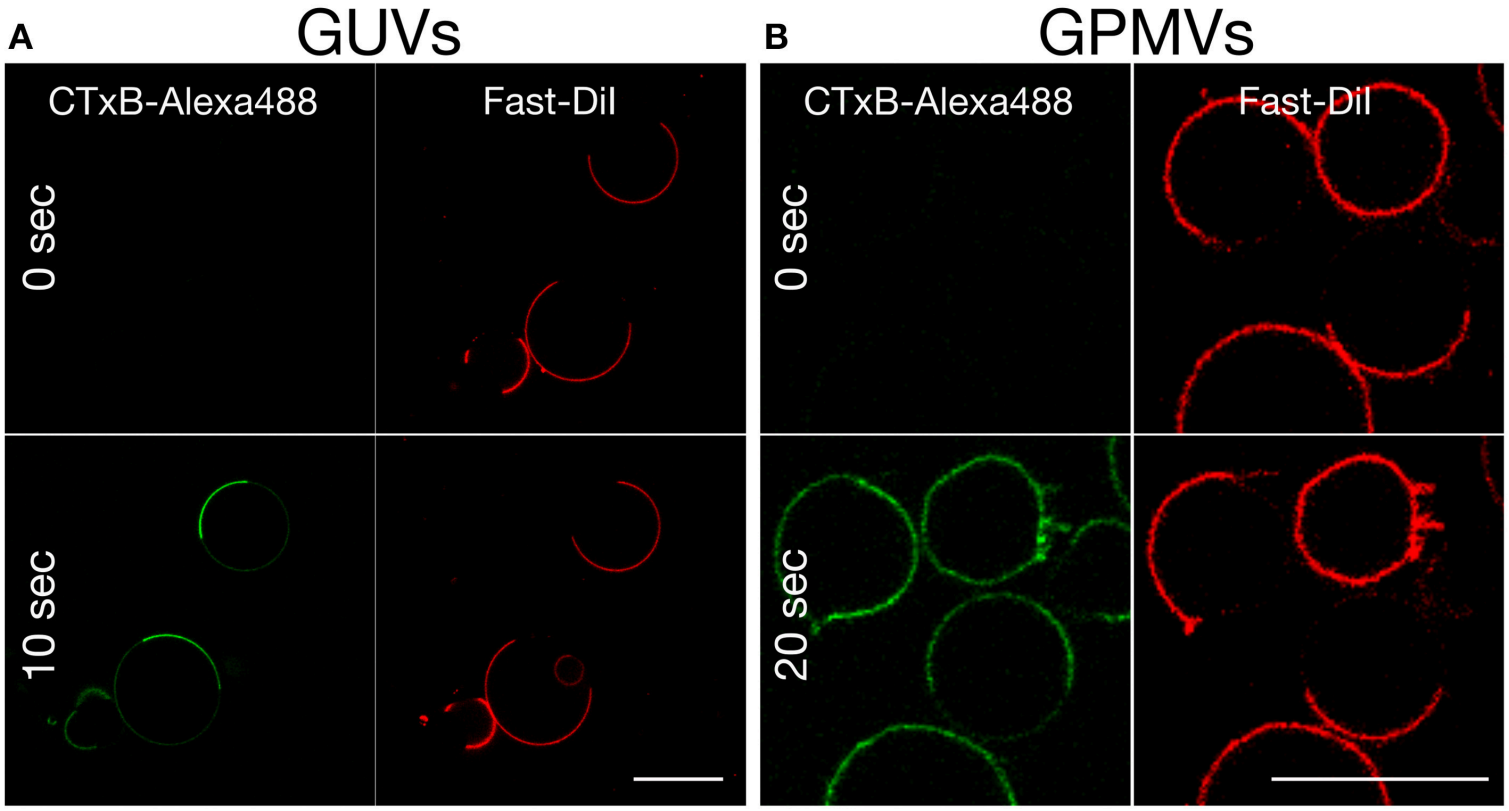
**Time-lapse confocal imaging of the equatorial plane of (A)** phase separated GUVs (DOPC:SSM:Chol:GM1 = 40:40:20:0.1) and **(B)** phase separated GPMVs prepared from RBL cells (labeled with the Ld marker Fast-DiI, red), following the addition (at time 0) and rapid binding of Alexa 488-labeled CTxB (green) to GM1. Binding occurs right after CTxB addition in the Lo phase. Scale bar 10 μm. Experiments were done at 10°C. Miscibility temperature for GPMVs is around 15°C.

The high specificity of CTxB binding to GM1 is commonly used to detect the presence of raft or Lo domains in cellular and synthetic membranes, respectively. However, based on our MD simulation data, slightly more efficient binding of CTxB to GM1 in the Ld environment is expected. One possible explanation for the discrepancy is that CTxB would bind GM1 preferentially in an Ld domain, but then the complex would move into an Lo domain, as suggested previously (Bacia et al., 2005). To test this possibility, we followed the binding of Alexa-labeled CTxB to GM1-containing GUVs and GPMVs immediately after addition of CTxB. In both model membranes, CTxB bound rapidly and largely to the Lo phase without obvious initial binding to the Ld phase (Figures 3A,B and Supplementary Movies 1, 2). Thus, it is unlikely that the experimentally observed binding to the Lo phase is a result of initial/stronger recognition of the GM1 molecules in the Ld phase by CTxB and subsequent partitioning of the GM1-CTxB complex into the Lo phase.

**Figure 4:**
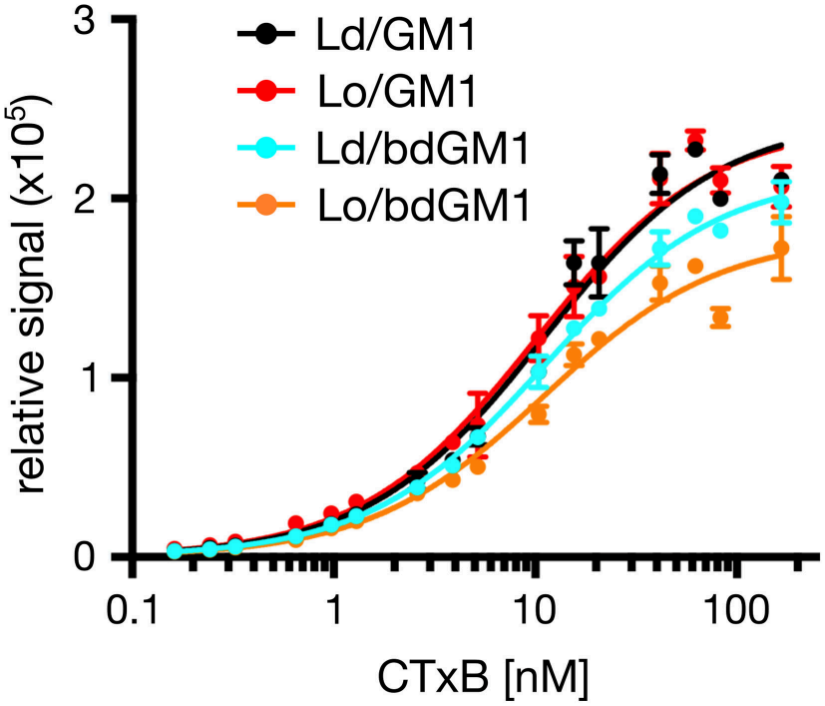
**Representative plot showing binding of Alexa 594-labeled CTxB to liposomes containing 0.1 mol % GM1 or bdGM1**.

An alternative explanation for the observed discrepancy is that CTxB binds its lipid receptor GM1 preferentially in Lo domains, once coexisting Ld/Lo phases are available. In order to selectively monitor CTxB binding to its lipid receptor in pure Ld or Lo membrane only, we used synthetic liposomes consisting of either DOPC (Ld) or SM/Chol (Lo) and 0.1 mol% of either GM1 or bdGM1. These liposomes were then used as capture specimen in electrochemiluminescence ELISA assays to quantitatively evaluate CTxB binding (Kolondra et al., 2010; Lingwood et al., 2011). Equal GSL content in liposomes was validated by thin-layer chromatography (TLC) analysis (Figure S6). In all systems we observed similar equilibrium binding affinities of ~15 nM, independent of lipid composition or GM1 acyl bodipy modification (Figure 4, and Table 1). However, we observed obvious differences in Bmax (maximum binding capacity) values. In Ld liposomes, bdGM1 showed Bmax to decrease ~20% compared to native GM1. In Lo domains, the decrease was ~35–40%. In our experimental conditions this means that less CTxB were bound to the bdGM1 vesicles than to those containing GM1. Since in all the studied systems the GM1 amounts were similar, the decreased Bmax values suggest that in the respective systems, a number of bdGM1 molecules remain hidden from CTxB. Although these observations are consistent with the trends predicted by atomistic MD simulations (Figures 2B,C), there is reason to keep in mind that the headgroup angle distribution and SASA of GM1 and bdGM1 showed only slight differences, and therefore the results suggest that other factors, e.g., ganglioside oligomerization may be involved (Shi et al., 2007).

**Table 1:** **Results for characteristics of Alexa594-labeled CTxB binding to liposomes containing 0.1 mol% GM1 or bdGM1 (*n* = 3)**.

**CTxB-Alexa594**	**Kd [nM]**	**Bmax**
Ld+GM1	16.56 ± 6.11	97.95% ± 1.93
Lo+GM1	15.59 ± 5.80	100%
Ld+bdGM1	13.48 ± 5.34	79.76% ± 7.23
Lo+bdGM1	14.85 ± 4.06	63.76% ± 10.52

*The Bmax was normalized to the highest value in each experiment*.

Our simulations and experimental measurements document that, although GM1 headgroup conformation is slightly modulated by the surrounding lipid environment, it is not the discriminating factor for the differences in CTxB binding to GM1 and its fluorescent analog. CTxB binding to GM1 shows a clear preference for the Lo phase in both GUVs and GPMVs (Figure 3), which in respect to slight headgroup conformation changes and comparable CTxB binding affinities to these molecules can only be explained by the enrichment of the GM1 lipids in the ordered phases (Morrow et al., 1995; Simons and Ikonen, 1997).

## Discussion and concluding remarks

Our atomistic MD simulation data confirmed that GM1 headgroup localization and geometry is sensitive to membrane environment (Figures 1, 2). Surprisingly, the presence of the bodipy-label at the acyl chain of GM1 caused a deeper penetration of bdGM1 into the membrane in the Lo domains (Figures 1C,E), demonstrated by decreased SASA and the density distribution of the forefinger structure (Figures 1B–E and Figures S2, S4, S5). The overall changes could thus render the bdGM1 headgroup partially inaccessible to CTxB. Our observations are consistent with previously reported NMR analysis in synthetic membranes, which showed that conformation and motional order of the complex ganglioside headgroups is influenced by factors such as natural variation in the glycolipid hydrocarbon chains, membrane fluidity, temperature, or the presence of cholesterol (Barber et al., 1994; Morrow et al., 1995; Singh et al., 1995). Interestingly, as presented by our simulations, the observed changes in GM1 headgroup conformation indicate a preference for CTxB binding to GM1 in the Ld environment rather than Lo (Figure 2). This result is in line with previously published atomistic MD simulations of GM1 in POPC or POPC/Chol membranes, which showed that GM1 headgroup adopts a tilted conformation in the presence of cholesterol, resulting in decreased recognition by CTxB (Lingwood et al., 2011). However, the effect of membrane phase on CTxB-GM1 interaction was weak in the present simulations, and this lack of a strong effect was validated experimentally by measuring binding of CTxB to synthetic liposomes in various phases (Table 1 and Figure 4). The binding data showed that the overall binding of CTxB to bdGM1 was reduced in comparison to GM1 especially in the Lo liposomes (Figure 4). Our data therefore confirm at the molecular level that the presence of the fluorophore on the acyl chain, rather than changing the headgroup geometry, largely excludes the bdGM1 molecules from the Lo membrane environments, where native GM1 molecules seemed to be enriched (Komura et al., 2016). Therefore, CTxB binding to GM1 occurs more in the ordered membranes, while binding to bdGM1 takes place preferably, if not exclusively, in the disordered domains (Sezgin et al., 2012b, 2015). The conclusion that the partitioning of bdGM1 does not properly represent the phase partitioning of GM1 is consistent with recent findings by Fricke and Dimova (Fricke and Dimova, 2016) published during the review of this paper.

Our experiments reveal that the recognition of bdGM1 by CTxB is decreased in comparison to native GM1 (Figure 4). Meanwhile, the MD simulations do not suggest strong structural changes within the headgroup region of the studied GM1 molecules. Therefore, it seems evident that the observed differences in CTxB recognition do not stem primarily from differences in headgroup conformation but rather from other factors such as clustering of GM1 lipids (Shi et al., 2007; Sachl et al., 2015). Consideration of such dynamical processes based on slow lateral diffusion through atom-scale MD simulations remains to be done in future work.

Gangliosides are important receptors at the cell surface, and the use of fluorescently labeled analogs is a common tool to study their cellular functions (Ewers et al., 2009; Sachl et al., 2015; Sezgin et al., 2015; Fricke and Dimova, 2016; Komura et al., 2016). However, the data presented here and in our previous work (Sezgin et al., 2012b) reveal that the presence of the fluorophore affects the behavior of the host lipid molecules. We have shown that acyl-chain labeling of GM1 changes its phase partitioning without strongly affecting ligand binding (here, CTxB). These effects should be considered for proper interpretation of cellular studies employing these and other fluorescent lipid analogs.

## Author contributions

All authors designed the experiments. SR, AO, TR, and OC performed the simulations. ES and MG performed the experiments. All authors contributed to the preparation of the manuscript.

## Funding

ES is supported by EMBO Long Term and Marie Curie Intra-European Fellowships (MEMBRANE DYNAMICS). MG and UC have been funded by the German Federal Ministry of Education and Research (BMBF) grant to the German Center for Diabetes Research (DZD e.V.). IV, TR, and SR acknowledge the Academy of Finland (Center of Excellence program) and the European Research Council (Advanced Grant CROWDED-PRO-LIPIDS) for financial support. SR thanks FEBS for the Short-Term Fellowship, the Graduate School program of Tampere University of Technology, and Alfred Kordelin Foundation for financial support. CE and ES are supported by the Wolfson Foundation (ref. 18272), the Medical Research Council (MRC, grant number MC_UU_12010/unit programmes G0902418 and MC_UU_12025), MRC/BBSRC/ESPRC (grant number MR/K01577X/1), and the Wellcome Trust (grant ref. 104924/14/Z/14).

### Conflict of interest statement

The authors declare that the research was conducted in the absence of any commercial or financial relationships that could be construed as a potential conflict of interest.
