# Supplementary material for: Phase Partitioning of GM1 and Its Bodipy-Labeled Analog Determine Their Different Binding to Cholera Toxin
Source: Front Physiol. 2017 May 9;8:252. doi: 10.3389/fphys.2017.00252 (PMC5422513; doi:10.3389/fphys.2017.00252)
Supplement: Supplementary file 1 [file Presentation1.PDF]

Supplementary Information

Phase Partitioning of GM1 and Its Bodipy-Labeled Analog Determine Their Different Binding to Cholera Toxin

Sami Rissanen[1,#], Michal Grzybek[2,3,#], Adam Orłowski[1,4], Tomasz Róg[1,5], Oana Cramariuc[1], Ilya Levental[6], Christian Eggeling[7], Erdinc Sezgin[7,*], Ilpo Vattulainen[1, 5, 8,*]

[1] Department of Physics, Tampere University of Technology, P.O. Box 692, FI-33101 Tampere, Finland

[2] Paul Langerhans Institute Dresden of the Helmholtz Centre Munich at the University Clinic Carl Gustav Carus, TU Dresden, 01307 Dresden, Germany

[3] German Center for Diabetes Research (DZD e.V.), 85764 Neuherberg, Germany

[4] Department of Physics and Energy, University of Limerick, Limerick, Ireland

[5] Department of Physics, P.O. Box 64, FI-00014 University of Helsinki, Finland

[6] Department of Integrative Biology and Pharmacology, University of Texas Health Science Center, United States

[7] MRC Human Immunology Unit, Weatherall Institute of Molecular Medicine, University of Oxford, UK

[8] MEMPHYS – Center for Biomembrane Physics, University of Southern Denmark, Odense, Denmark

[#] These authors contributed equally

* To whom correspondence may be addressed:
Erdinc Sezgin (erdinc.sezgin@rdm.ox.ac.uk) and Ilpo Vattulainen (ilpo.vattulainen@helsinki.fi).

Supplementary Information: Methods and Materials

Molecular Dynamics Simulations

We performed atomistic molecular dynamics (MD) simulations of eight systems from which 4 were comprised of a lipid bilayer and Cholera Toxin B-pentamer (PDB record: 1RF2); lipid compositions represented the liquid-disordered (Ld) and liquid-ordered (Lo) phases. In both cases 8 mol% bdGM1 or GM1 was added to the bilayer. Additionally, we simulated 4 lipid bilayers as reference systems with the same composition as for the systems

with the protein (detailed composition of all simulated systems is shown in **Table S1**). The systems were hydrated with approximately 50,000 or 20,000 water molecules (for systems with and without the protein, respectively).

For the first four systems Cholera Toxin B-pentamer was positioned in water above the membrane. First, the distance between the center of mass (COM) of the protein and the COM of the membrane varied between 6.6 – 7.1 nm, depending on the system. Moreover, the minimum distance between the membrane and the protein was about 1.0 - 1.5 nm, depending on the system. The initial structure is visualized in **Figure 2A**. When the simulations were started, the protein was placed above the membrane in a manner where the CTxB binding sites for GM1 pointed down towards the membrane. Therefore, the starting structures used in the simulations were based on experimental data and were therefore consistent with experiments. For the binding site based on the crystal structure, see e.g. the PDB ID: 2XRQ. Further, for each of the systems we carried out three independent simulations (**Table S1**). In these simulations, as described below in more detail, the starting structure of the membrane was made different by shuffling the lipid organization in the membrane plane, and the distance and the orientation of the protein with respect to the membrane varied from one simulation to another. Through these changes, the protein interacted with the membrane in a different manner in the different simulations. Despite these differences, for each of the studied systems the results of the three simulations were consistent with each other.

In the simulations, NaCl ions were added to result in 140 mM NaCl solution. The first four systems (with the protein) were simulated for 1000 ns and the remaining ones (pure bilayers) were simulated for 200 ns, both using the GROMACS 4.5.5 software package (1). The OPLS all-atom force field (2) was used to parameterize all molecules. For water, we employed the TIP3P model that is compatible with the OPLS parameterization (3). The system setup used in this study is identical to that used in our previous simulations of lipid bilayers with the OPLS-AA parameterization (4, 5) with bodipy label parametrization details and methods provided below. Periodic boundary conditions with the usual minimum image convention were used in all three directions. The LINCS algorithm (6) was used to preserve the length of each hydrogen atom covalent bond. The time step was set to 2 fs and the simulations were carried out at a constant pressure (1 bar) and temperature (298 K). The temperature and pressure were controlled by the Parrinello-Rahman and v-rescale methods, respectively (7, 8). The temperatures of the solute and solvent were controlled independently. For pressure, we used a semi-isotropic control for the systems with bilayer and isotropic for the peptide in water. The Lennard-Jones interactions were cut off at 1.0 nm, and for the electrostatic interactions we employed the particle mesh Ewald method (9) with a real space cutoff of 1.0 nm, beta-spline interpolation (order of 6), and direct sum tolerance of 10^{-6}. Pictures were made with the VMD package. For completeness, data for the average area per lipid are shown in **Table S7**.

Dependence of GM1 Headgroup Conformation on Lipid Content

To consider how the GM1 headgroup orientation depends on cholesterol content and the lipid composition overall, we also simulated a system comprised of DOPC, cholesterol, and GM1 (system 9 in **Table S1**). The DOPC/Chol/GM1 ratios used were 220/220/40 molecules, therefore matching the ratios used in SSM/Chol/GM1 systems.

Parametrization of Bodipy

Geometry optimizations were carried out by employing the density functional theory (DFT) method with the B3LYP (10) functional and the 6-31G* basis set. The Gaussian 09 electronic structure program was used (11). The ChelpG atomic charges and dipoles were calculated from the electronic density implementing the ChelpG(12) routine of the Gaussian package imposing the restriction that the ChelpG molecular dipole moment reproduces the DFT/B3LYP/6-31* molecular dipole moment calculated directly from the electronic density. The

hybrid density functional method B3LYP was previously shown to give charges of the same quality as correlated *ab initio* methods(13). The default values were used, including point densities and atomic radii. Charges on equivalent atoms were set to be the same. The resulting charges are shown in **Figure S7**.

Topologies and Simulation Parameters for GM1

For completeness, we provide the topology, structure, and simulation parameter files of GM1 as a separate supplementary file (Supplementary Data File).

Calculation of Solvent Accessible Surface Area from Simulation Data

The solvent accessible surface area (SASA) refers to the surface area of a molecule that is accessible to solvent and is defined as a part of the surface of a sphere centered at an atom with radius $r_{VDW} + r_{SOL}$, where the center of a spherical solvent molecule or probe (r_{SOL}) can be placed to be in contact with the atomic van der Waals sphere (r_{VDW}) without penetrating other atoms (14). For a molecule, it is the sum over all of its atoms. In this work, SASA was computed with the Gromacs tool `gmx_sasa`.

Materials

Cholesterol, N-stearoyl-D-erythro-sphingosylphosphorylcholine (SSM), 1,2-dioleoyl-sn-glycero-3-phosphocholine (DOPC), and GM1 were from Avanti; BODIPY® FL C_5-ganglioside GM1 (bdGM1) was purchased in Thermofisher; Alexa (488 and 594)-labeled-cholera toxin B subunit were from Invitrogen; mouse anti-cholera toxin b antibodies were from USBiologicals; goat anti-mouse- STAG antibodies and plates for electrochemiluminescence (EIA) assay were from Meso Scale Discovery;

Liposome Preparation and Cholera Toxin B Binding

The EIA experiments were performed according to the manufacturer's protocol (Meso Scale Discovery, MSDH) based on the sandwich immunoassay, which utilizes electrochemiluminescence to measure protein levels (15). Briefly, liposomes containing 0.1 mol% of either GM1 or bdGM1 were prepared by mixing the organic solutions of the respective lipids. The mixtures were dried under nitrogen stream and left under vacuum for 1h. Rehydration of 1 mg dried lipids film was done in 1 mL buffer (25 mM HEPES, 150 mM NaCl, pH 7.25). The resulting multilamellar vesicles were subsequently subjected to 10 freezing/thawing cycles (liquid nitrogen/60 deg) and extruded through 100 nm polycarbonate filters. Liposomes were passively adsorbed on the electrode surface (1 h, 23 deg), and the residual sites on the surface were blocked with 0.2 % porcine gelatin (1 h, 23 deg). The surface was then washed three times with buffer and porcine gelatin solutions containing the desired concentrations of alexa594-labeled CTxB (were added to each well). Binding was carried out for 2 h at 23 deg. Wells were then washed and a solution of primary antibody against the CTxB was applied (1 µg/mL, 23 deg, 1 h). The wells were again washed, and secondary antibody (goat anti-mouse- STAG) was added (1 µg/mL, 23 deg, 1 h). The wells were washed and reading buffer was added (MSD surfactant-free reading buffer). The background was determined from binding of both primary and secondary antibodies to liposomes. Data were acquired on a SECTOR Imager 6000. The recorded data were analyzed using GraphPad Prism 6.0 software using one site-specific binding algorithm.

Thin layer chromatography

Liposomes were extracted using a two-step extraction protocol using 3 volumes of chloroform/methanol 10:1, followed by 3 volumes of chloroform/methanol 2:1. The lipid-containing organic phases from both steps were pooled and dried under a nitrogen stream. For thin layer chromatography (TLC) analysis, lipids were dissolved in 30 µl of chloroform/methanol (2:1) and applied to a TLC plate (HPTLC Silica Gel 60, Merck). A 60:35:8

mixture of chloroform/methanol/CaCl2 (0.2% CaCl2 solution) was used as a running phase for TLC analysis. The lipids were visualized by spraying the plate with copper sulphate solution (3% Copper Sulphate in 15% phosphoric acid) and heating (200 C, 3–5 min).

GUV Preparation
Small amount of 1 mg/ml lipid mixtures were spread onto a platinum wire which was then dipped into 300 mM sucrose solution in a Teflon chamber. GUV formation was triggered with 2 V 10 Hertz electric current for 1 h, at 68 deg followed by 2 Hz at room temperature.

GPMV Preparation
After 70-80 % cells confluency, GPMVs were isolated by chemically inducing cell blebbing with paraformaldehyde and DTT (dithiothreitol) containing buffer (150 mM NaCl, 10 mM Hepes and 2 mM CaCl2 (pH 7.4)) for 2 h at 37 deg as previously described. The fluorescent lipids or dyes were added to GPMVs at a concentration of 0.1-1 μM. For imaging phase separation, vesicles were cooled to 10 deg with a microscope-mounted Peltier element.

Fluorescent lipids were added into the GPMV suspension, and due to their lipophilicity they very efficiently got incorporated into the GPMVs. Being more precise, fluorescent lipids were dissolved either in ethanol or DMSO and they were added into the vesicle suspension using a very small concentration (1 μl to 1000 μL), thus the effect of solvent was not notable (or in any way significant).

Confocal Imaging and Partitioning Measurements
GUVs and GPMVs were imaged with a Zeiss LSM 780 confocal microscope in BSA-coated Labtek chambers. 488 nm, 543 nm and 633 nm lasers were used for excitation of green, orange and red fluorophores, respectively. BP 530-550, BP 585-615 and LP 650 filters in multi-track mode were used to eliminate the cross talk.

ImageJ-Line profile was used to calculate the Lo %. A line was selected which crosses the opposite sides of the equatorial plane of the GUVs or GPMVs having different phases on opposite sides. Opposite sides are chosen to eliminate the laser excitation polarization artefacts. Then, %Lo was calculated as

$F_{Lo}/(F_{Lo}+F_{Ld})$ (Eq. 1)

where F is the fluorescence emission intensity. If *%Lo* > 50 %, a lipid analog prefers the liquid ordered (or raft) phase.

GM1 Incorporation
GM1 or bdGM1 was dissolved in Ethanol (1 mg/ml). 1:200 of this suspension was added to the cells in serum-free media (5 μg/ml final concentration). Cells were incubated with bdGM1 for 5 minutes and with GM1 for 1 hour at 37 deg.

Cholera Toxin B Treatment
Alexa-labeled CTxB was incubated with the model membranes at room temperature for 15 minutes and cells at 37 deg for 10 minutes (5 nm final concentration).

The mixing of CTxB with the GUVs and GPMVs and the observation was carried out in real time on the microscope. The imaging chamber (ibidi 8-well chamber) was an open imaging chamber, therefore addition of small volumes was possible. Time-lapse imaging started before the addition of CTxB. The supplementary movies show the moment when CTxB was gently added in a manner where the field of view remained the same.

In this fashion, one can observe the time t = 0, which is the time CTxB was added.

Comparison of Bmax with Simulation Data

Bmax is the maximum number of binding sites (i.e., the number of receptors) available at the surface. In case of CTxB, which is a pentamer, the strongest binding would be obtained if all binding pockets would be occupied (meaning that each pentamer would bind 5 GM1 molecules), however the highest Bmax would be obtained if each pentamer would bind only one GM1 molecule. Since in the MD simulations we observed quite similar binding characteristics of CTxB to GM1 in Ld and Lo environments, the enhanced fluorescence intensity in the Lo phase of GUVs is most probably caused by preferential partitioning of GM1 molecules to that given phase.

Supplementary Information: Tables

Table S1. Description of the simulated systems including data for the number of molecules in each system and simulation times of each system. The key simulations (systems 1-4) have been repeated in 3 replicas each.

System	CTxB	DOPC	SSM	Chol	bdGM1	GM1	Water	Na^+	Cl^-	Simulation time (nanoseconds)
1	1	440	0	0	40	0	65588	218	183	3 x 1000
2	1	0	220	220	40	0	46128	164	129	3 x 1000
3	1	440	0	0	0	40	62206	208	173	3 x 1000
4	1	0	220	220	0	40	43678	156	121	3 x 1000
5	0	440	0	0	40	0	20314	97	57	200
6	0	0	220	220	40	0	14844	81	41	200
7	0	440	0	0	0	40	20044	96	56	200
8	0	0	220	220	0	40	13947	79	39	200
9	0	220	0	220	0	40	13947	79	39	2 x 1000

Table S2. SASA values per lipid molecule. Values were calculated as an average over lipids from the leaflet where CTxB is not binding (systems 1-4, **Table S1**).

System	Lipid	SASA per lipid (nm^2)
Ld/GM1	GM1	6.628 ± 0.092
Lo/GM1	GM1	6.485 ± 0.078
Ld/bdGM1	bdGM1	6.729 ± 0.007
Lo/bdGM1	bdGM1	6.295 ± 0.096
Ld/GM1	DOPC	1.356 ± 0.014
Lo/GM1	SSM	1.673 ± 0.005
Ld/bdGM1	DOPC	1.361 ± 0.016
Lo/bdGM1	SSM	1.734 ± 0.015

Table S3. Average angles between bilayer normal and vectors describing GM1 and bdGM1 headgroup components as shown schematically in **Figure 1B**. Values were calculated as an average over all GM1 lipids from the leaflet where CTxB is not binding (systems 1-4, **Table S1**). Simulation time period of 300-1000 ns was used in the calculations. Error bars correspond to standard error.

Vector	System	Average angle (°)
Forefinger	Ld/GM1	93.90 (± 1.84)
Forefinger	Ld/bdGM1	97.04 (± 1.40)
Forefinger	Lo/GM1	92.37 (± 2.44)
Forefinger	Lo/bdGM1	92.95 (± 2.00)
Thumb	Ld/GM1	73.47 (± 1.69)
Thumb	Ld/bdGM1	70.40 (± 1.44)
Thumb	Lo/GM1	71.45 (± 2.45)
Thumb	Lo/bdGM1	73.25 (± 2.15)
Wrist	Ld/GM1	29.91 (± 1.01)
Wrist	Ld/bdGM1	25.20 (± 0.91)
Wrist	Lo/GM1	31.24 (± 1.64)
Wrist	Lo/bdGM1	26.59 (± 1.21)

Table S4. Average angles between bilayer normal and the GM1 and bdGM1 headgroups' forefinger rings vectors and two wrist vectors as shown schematically in **Figure 1B**. Values were calculated as an average over all GM1 lipids from the leaflet where CTxB is not binding (systems 1-4, **Table S1, Table S3**). Simulation time period of 300-1000 ns was used in the calculations. Error bars correspond to standard error.

Vector	System	Average angle (°)
Forefinger middle ring	Ld/GM1	77.83 (± 1.67)
Forefinger middle ring	Ld/bdGM1	82.32 (± 1.27)
Forefinger middle ring	Lo/GM1	78.67 (± 2.51)
Forefinger middle ring	Lo/bdGM1	77.27 (± 1.79)
Forefinger top ring	Ld/GM1	89.70 (± 2.00)
Forefinger top ring	Ld/bdGM1	92.55 (± 1.61)
Forefinger top ring	Lo/GM1	88.56 (± 2.52)
Forefinger top ring	Lo/bdGM1	88.95 (± 2.03)
Wrist long	Ld/GM1	45.00 (± 1.54)
Wrist long	Ld/bdGM1	47.73 (± 1.07)
Wrist long	Lo/GM1	46.59 (± 2.32)
Wrist long	Lo/bdGM1	44.60 (± 1.70)
Wrist middle	Ld/GM1	28.00 (± 1.09)
Wrist middle	Ld/bdGM1	25.16 (± 0.67)
Wrist middle	Lo/GM1	29.78 (± 1.73)
Wrist middle	Lo/bdGM1	25.08 (± 1.20)

Table S5. Average number (during a period of 300–1000 ns) of hydrogen bonds between GM1 and CTxB residues (systems 1-4, **Table S1)**. Residues found to be responsible for binding to GM1 (see *E. A. Merritt et al., Crystal structure of cholera toxin B-pentamer bound to receptor GM1 pentasaccharide, Protein Science* ***3****, 166–175 (1994)*) depicted with *. Only the residues forming more than 0.1 hydrogen bonds are shown. Error bars correspond to standard error.

	**Ld/GM1**	**Lo/GM1**	**Ld/bdGM1**	**Lo/bdGM1**
**Thr1**	0.265 (± 0.244)	1.571 (± 0.957)	1.912 (± 0.383)	0.286 (± 0.156)
**Gln3**	–	–	0.136 (± 0.132)	0.027 (± 0.027)
**Glu11***	0.176 (± 0.008)	0.485 (± 0.197)	0.533 (± 0.241)	0.192 (± 0.112)
**Tyr12**	0.141 (± 0.132)	0.091 (± 0.059)	0.011 (± 0.007)	0.052 (± 0.050)
**His13***	0.756 (± 0.364)	0.467 (± 0.231)	0.438 (± 0.053)	0.453 (± 0.193)
**Asn14**	0.593 (± 0.371)	0.885 (± 0.497)	0.346 (± 0.067)	0.333 (± 0.208)
**Thr15**	0.200 (± 0.200)	0.003 (± 0.003)	0.005 (± 0.004)	0.033 (± 0.033)
**Gln16**	0.036 (± 0.025)	0.128 (± 0.084)	0.154 (± 0.079)	0.121 (± 0.117)
**Gly33**	0.032 (± 0.018)	0.012 (± 0.011)	0.149 (± 0.100)	0.252 (± 0.246)
**Lys34**	4.325 (± 0.492)	2.317 (± 0.621)	1.288 (± 0.527)	1.564 (± 0.398)
**Arg35**	3.193 (± 1.409)	1.262 (± 1.639)	2.254 (± 0.749)	0.317 (± 0.173)
**Glu51***	0.042 (± 0.028)	0.172 (± 0.128)	0.463 (± 0.463)	0.002 (± 0.001)
**Gly54**	0.325 (± 0.279)	0.112 (± 0.068)	0.009 (± 0.004)	0.001 (± 0.001)
**Ser55**	2.237 (± 0.896)	0.777 (± 0.265)	0.648 (± 0.457)	0.798 (± 0.281)
**Gln56***	0.857 (± 0.586)	1.057 (± 0.300)	0.650 (± 0.314)	0.996 (± 0.590)
**His57**	0.295 (± 0.198)	0.270 (± 0.212)	0.287 (± 0.143)	0.168 (± 0.090)
**Ile58**	0.022 (± 0.013)	0.066 (± 0.038)	0.048 (± 0.028)	0.341 (± 0.341)
**Asp59**	0.720 (± 0.285)	0.375 (± 0.142)	0.360 (± 0.360)	0.940 (± 0.323)
**Ser60**	0.317 (± 0.195)	0.045 (± 0.031)	0.003 (± 0.003)	0.001 (± 0.001)
**Gln61***	0.204 (± 0.152)	0.110 (± 0.054)	0.070 (± 0.037)	0.018 (± 0.017)
**Lys62**	1.961 (± 0.455)	0.091 (± 0.088)	0.356 (± 0.354)	0.597 (± 0.439)
**Lys63**	0.124 (± 0.088)	0.265 (± 0.262)	0.091 (± 0.091)	1.082 (± 0.578)
**Trp88**	0.094 (± 0.082)	0.111 (± 0.097)	0.041 (± 0.040)	0.017 (± 0.016)
**Asn89**	0.335 (± 0.237)	0.366 (± 0.352)	0.174 (± 0.110)	0.095 (± 0.076)
**Asn90***	0.742 (± 0.352)	0.435 (± 0.224)	0.893 (± 0.443)	0.579 (± 0.546)
**Lys91***	0.843 (± 0.256)	0.709 (± 0.408)	1.069 (± 0.365)	0.944 (± 0.866)
**Thr92**	0.037 (± 0.034)	0.193 (± 0.159)	0.404 (± 0.217)	0.177 (± 0.139)
**TOTAL**	18.872 (± 0.274)	12.375 (± 0.227)	12.792 (± 0.214)	10.386 (± 0.223)

Table S6. Contacts between GM1 molecules and each of the protein residues during the simulations (systems 1-4). Only the residues in contact with the GM1 head group in the crystal structure (PDB record: 1RF2, marked as *) and the residues that have more than one contact during the simulations are shown. The contacts were calculated by using the distance cut-off of 0.35 nm between heavy atoms.

	**Ld/GM1**	**Lo/GM1**	**Ld/bdGM1**	**Lo/bdGM1**
**Thr1***	6.048 (± 4.094)	23.644 (± 13.608)	28.865 (± 5.101)	6.136 (± 2.773)
**Pro2***	0.453 (± 0.251)	0.852 (± 0.442)	0.625 (± 0.407)	0.559 (± 0.465)
**Gln3**	0.000 (± 0.000)	0.012 (± 0.010)	2.622 (± 2.535)	0.755 (± 0.632)
**Ala10**	3.241 (± 1.961)	1.214 (± 0.645)	1.968 (± 1.023)	0.532 (± 0.266)
**Glu11***	4.574 (± 0.186)	7.789 (± 1.563)	11.107 (± 6.174)	3.996 (± 1.851)
**Tyr12***	7.797 (± 3.110)	9.071 (± 6.080)	4.980 (± 1.417)	4.570 (± 0.317)
**His13**	34.213 (± 17.115)	29.088 (± 14.343)	30.406 (± 6.739)	17.795 (± 8.080)
**Asn14**	18.404 (± 9.565)	21.869 (± 12.421)	15.424 (± 5.655)	17.811 (± 0.389)
**Thr15**	3.676 (± 3.663)	1.402 (± 0.943)	0.220 (± 0.129)	1.233 (± 0.878)
**Gln16**	1.974 (± 1.318)	7.255 (± 5.593)	3.966 (± 1.973)	5.964 (± 4.680)
**Gly33***	2.912 (± 1.406)	1.003 (± 0.909)	3.798 (± 2.466)	3.753 (± 2.856)
**Lys34**	90.575 (± 10.791)	44.752 (± 14.036)	40.811 (± 11.552)	34.644 (± 9.115)
**Arg35***	51.242 (± 15.181)	24.431 (± 14.359)	38.819 (± 6.291)	15.726 (± 1.607)
**Glu36**	0.727 (± 0.353)	0.102 (± 0.060)	0.013 (± 0.011)	0.122 (± 0.121)
**Met37**	0.047 (± 0.041)	0.585 (± 0.532)	0.965 (± 0.965)	0.042 (± 0.041)
**Glu51***	5.377 (± 2.745)	3.864 (± 2.075)	9.216 (± 5.329)	0.493 (± 0.476)
**Val52**	7.528 (± 2.326)	6.198 (± 3.076)	12.333 (± 3.213)	0.728 (± 0.559)
**Pro53**	6.489 (± 2.482)	1.209 (± 0.553)	1.844 (± 0.688)	0.165 (± 0.061)
**Gly54**	17.051 (± 0.337)	8.648 (± 2.491)	8.234 (± 2.906)	2.705 (± 1.559)
**Ser55**	47.765 (± 6.673)	21.584 (± 6.513)	12.207 (± 4.102)	23.751 (± 5.840)
**Gln56***	38.688 (± 22.660)	52.079 (± 18.451)	22.346 (± 7.476)	54.617 (± 30.485)
**His57***	17.326 (± 6.815)	18.887 (± 5.704)	9.263 (± 4.420)	18.301 (± 11.661)
**Ile58**	49.378 (± 9.608)	22.832 (± 7.179)	11.882 (± 5.494)	39.722 (± 16.185)
**Asp59**	20.344 (± 7.319)	7.417 (± 1.718)	9.241 (± 9.237)	17.461 (± 3.472)
**Ser60**	13.183 (± 4.169)	1.155 (± 0.490)	0.543 (± 0.499)	0.192 (± 0.082)
**Gln61***	6.725 (± 4.502)	4.277 (± 1.872)	4.997 (± 2.545)	0.441 (± 0.251)
**Lys62**	30.634 (± 7.199)	1.913 (± 1.421)	9.013 (± 8.898)	11.014 (± 6.152)
**Lys63**	4.304 (± 2.010)	2.848 (± 2.317)	2.115 (± 2.114)	16.285 (± 10.091)
**Trp88***	9.358 (± 4.332)	13.937 (± 7.114)	10.511 (± 2.372)	6.213 (± 5.299)
**Asn89**	7.298 (± 3.812)	7.355 (± 6.602)	3.294 (± 2.168)	6.625 (± 4.234)
**Asn90***	19.349 (± 9.277)	18.019 (± 12.140)	17.893 (± 8.802)	20.454 (± 11.133)
**Lys91***	18.404 (± 3.187)	20.138 (± 2.573)	32.211 (± 5.414)	23.272 (± 20.976)
**Thr92***	2.155 (± 2.084)	7.602 (± 4.629)	10.066 (± 4.404)	7.080 (± 3.767)
**Pro93**	0.349 (± 0.230)	0.024 (± 0.011)	2.260 (± 1.522)	0.318 (± 0.176)
**Ala95**	1.172 (± 0.643)	1.099 (± 0.887)	0.377 (± 0.376)	0.000 (± 0.000)
**All**	548	394	374	363
**All***	190	205	204	165

Table S7. Area per lipid calculated over the time period of 100-200 ns in the systems 5-8. Area was calculated by dividing the total area of the simulation box by the number of lipids in a single bilayer leaflet. In systems with cholesterol, the area per cholesterol was assumed to be 0.38 nm^2 per molecule, which was first extracted from the total area (for discussion of various methods of calculation of the surface area of membranes with cholesterol, see *Biochim. Biophys. Acta.* 1788:97-121 (2009)). Error bars correspond to standard error.

System	Area per lipid (nm^2)
GM1 in Ld	0.62 (± 0.01)
bdGM1 in Ld	0.63 (± 0.01)
GM1 in Lo	0.42 (± 0.01)
bdGM1 in Lo	0.43 (± 0.01)

Supplementary Information: Figures

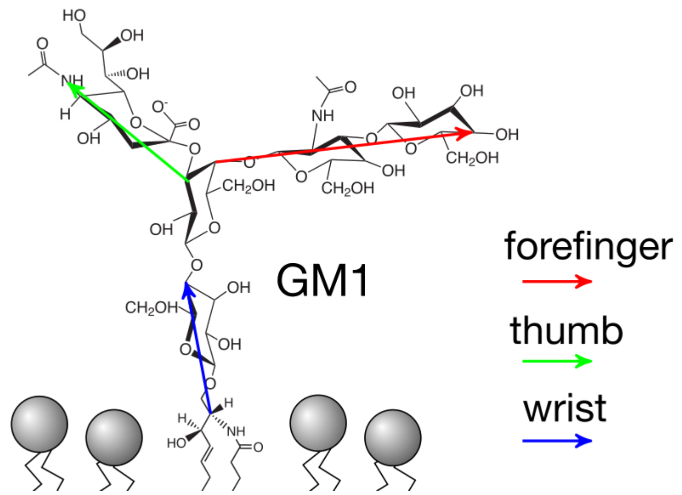

Figure S1. Illustration of the GM1 headgroup and its different domains, as expanded from **Figure 1B**.

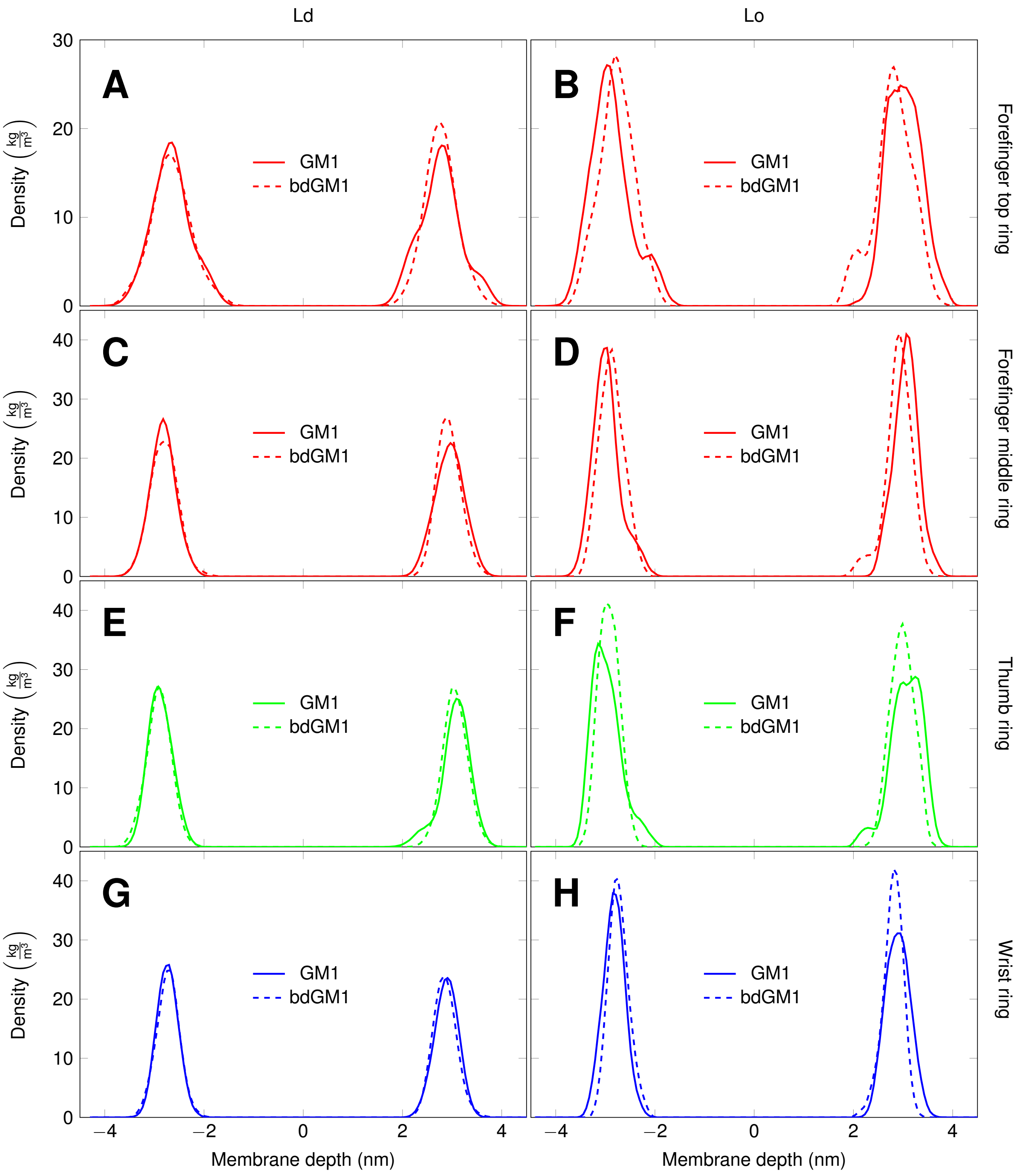

Figure S2. Density profiles of atoms (along the membrane normal direction in units of a nanometer) from headgroup sugar rings (as schematically shown in **Figure 1A, Table S3, Table S4**). Color code is as follows: bdGM1 – dashed lines; GM1 – solid lines in the Ld (A, C, E, G) and Lo phases (B, D, F, H); A, B – forefinger top ring; C, D – forefinger middle ring; E, F – thumb ring; G, H – wrist ring. Calculated from the systems 5-8 (**Table S1)** over a period of 100-200 ns. A slight shift of the atoms towards the membrane is observed in forefinger for bdGM1 in the Lo phase. Zero corresponds to the bilayer center.

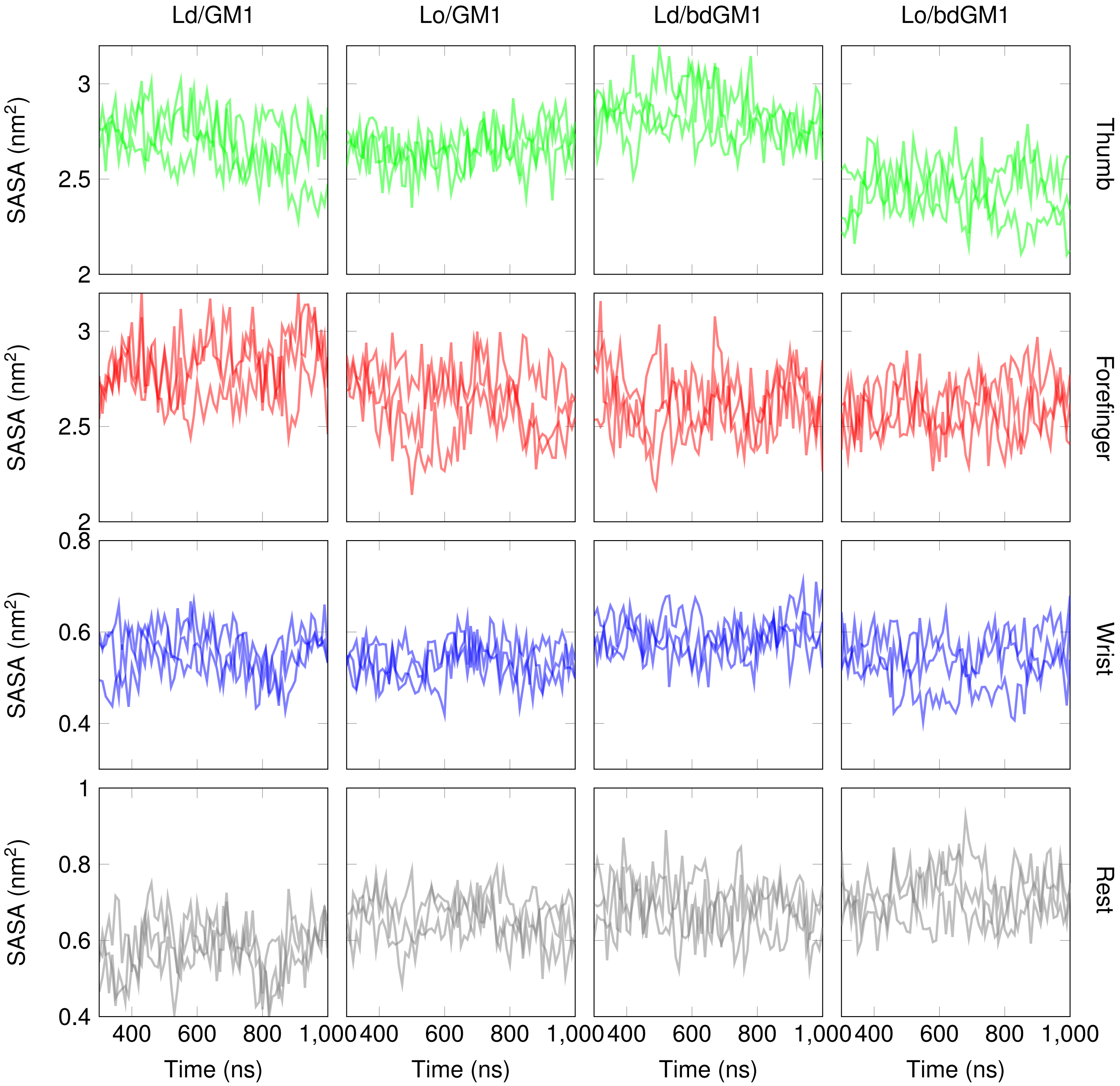

Figure S3. Solvent accessible surface area (SASA) calculated for different parts of GM1 and bdGM1 molecules using the `g_sasa` tool from the GROMACS simulation package. Total values are shown in **Figure 1**. In each graph, three replicas are shown by means of transparent lines.

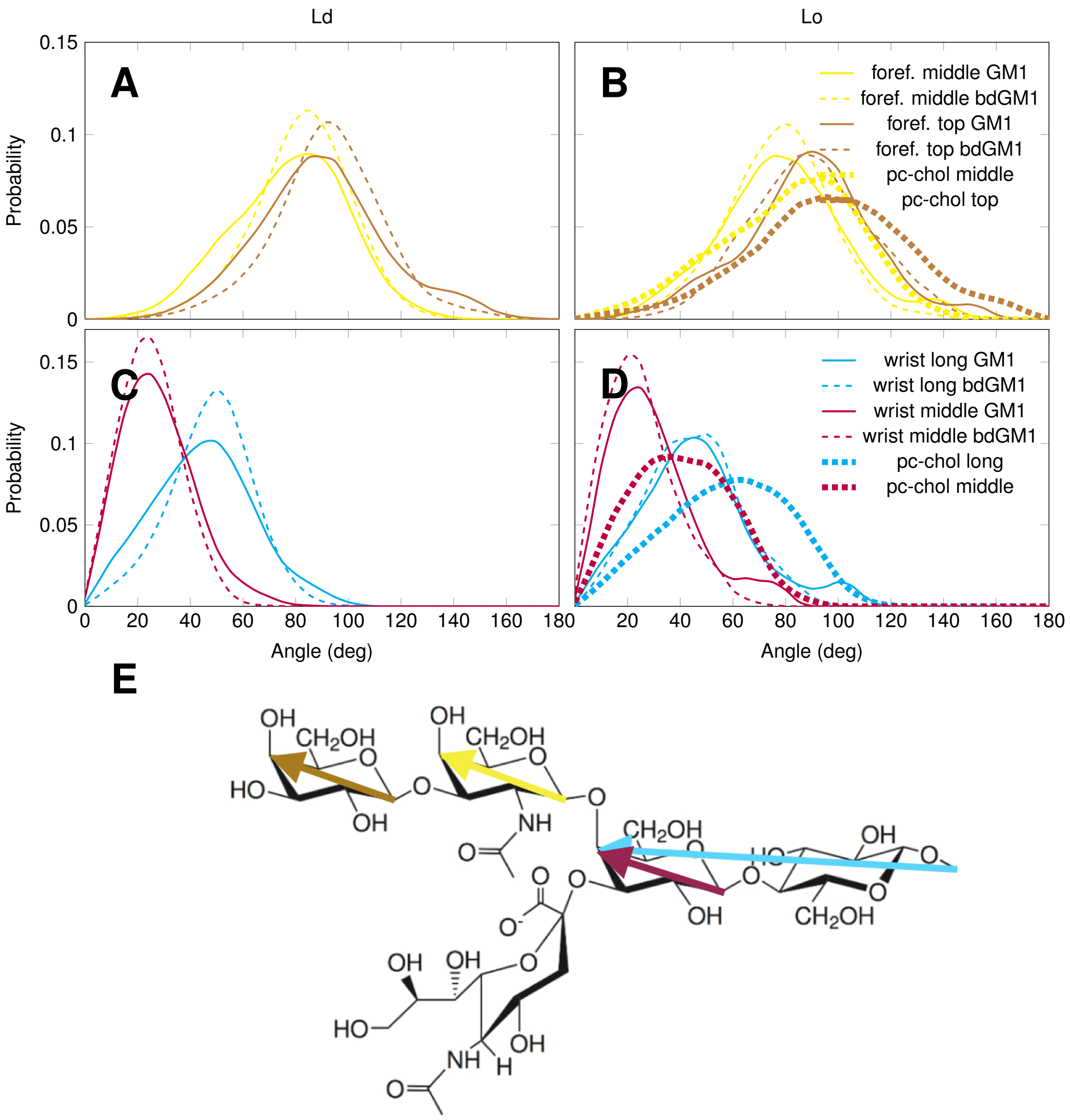

Figure S4. Distributions of angles between the bilayer normal and vectors representing forefinger's middle ring (yellow), forefinger top ring (brown), wrist middle (purple), and wrist long (cyan); (dashed lines – bdGM1 and solid lines - GM1) in Ld (A, C) and Lo (B, D) defined as schematically presented in (E) – colors of vectors correspond to colors of the lines in A-D. Data were calculated as an average over all GM1 lipids (systems 1-4, **Table S1**). Only the bilayer leaflet where CTxB is not binding was used. Simulation time period of 300-1000 ns was used in the calculations. Also given are the results for system 9 (see discussion in the text).

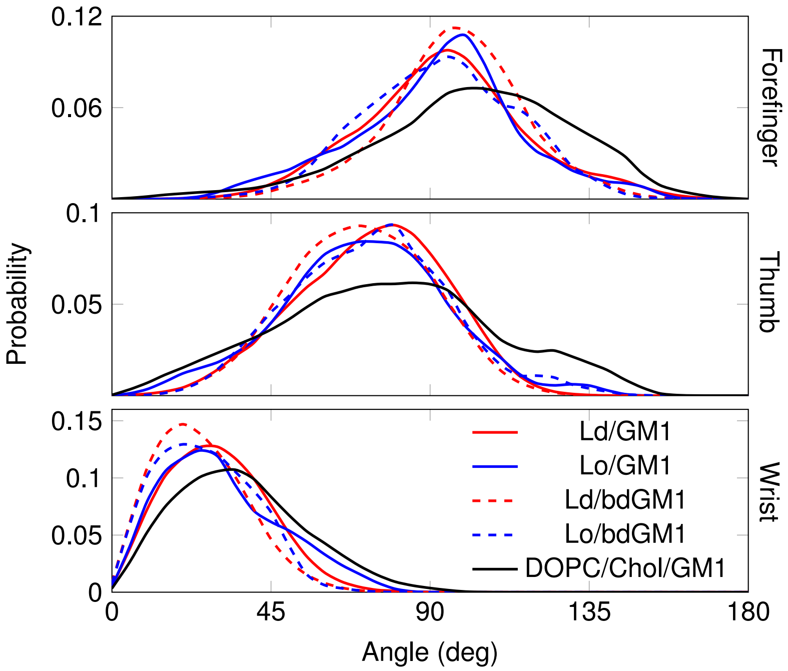

Figure S5. Results showing the tilt of the GM1 headgroup (separately for the forefinger, thumb, and wrist sections) in the four systems explored in the paper, and also for the DOPC-Chol system with GM1. One can find that in the DOPC-Chol system, the GM1 headgroup tilts considerably more towards the membrane plane compared to the other systems studied here.

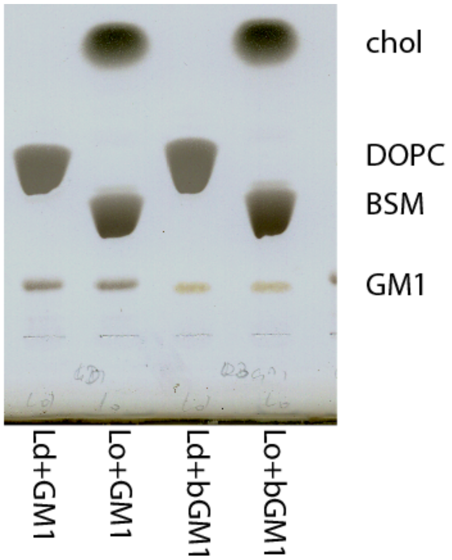

Figure S6. Thin layer chromatography comparing the lipid composition of liposomes used for the CTxB binding (Figure 4).

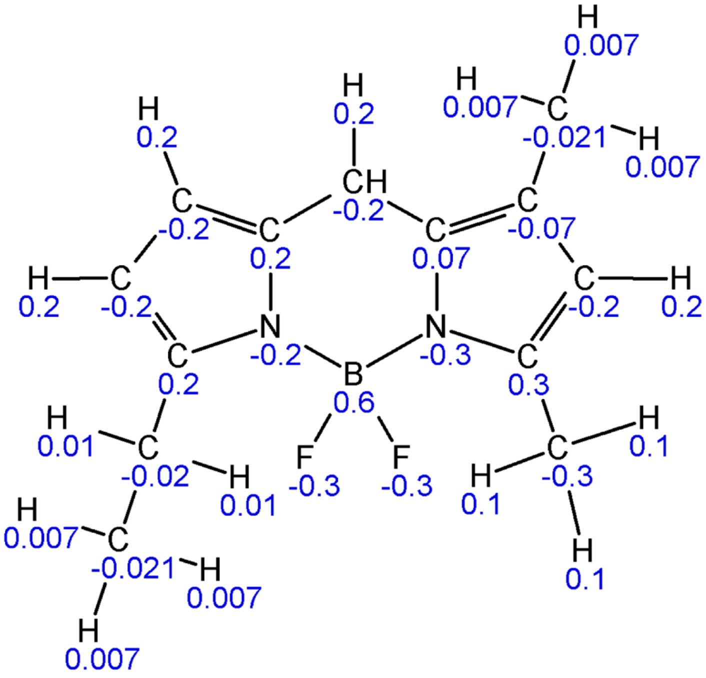

Figure S7. Two-dimensional representation of the bodipy molecular structure together with partial charges used in the simulation models.

Supplementary Data

Supplementary Data File: Topologies, structure, and simulation parameters for GM1.

Supplementary Information: Movies

Supplementary Movie 1: Time-lapse imaging of CTxB binding to phase separated GUVs. Alexa488-labelled CTxB (green channel) is added on GUVs with a final concentration of 400 ng/ml. Confocal images were taken every 5 seconds. Fast DiI (red channel) was used as a disordered domain marker.

Supplementary Movie 2: Time-lapse imaging of CTxB binding to phase separated GPMVs. Alexa488-labelled CTxB (green channel) is added on GPMVs with a final concentration of 400 ng/ml. Confocal images were taken every 5 seconds. Fast DiI (red channel) was used as a disordered domain marker.

Supplementary References

1. Hess B, Kutzner C, van der Spoel D, & Lindahl E (2008) GROMACS 4: Algorithms for highly efficient, load-balanced, and scalable molecular simulation. *J Chem Theory Comput* 4(3):435-447.
2. Jorgensen W & Tirado-Rives J (1988) The OPLS potential functions for proteins. Energy minimizations for crystals of cyclic peptides and crambin. *J Am Chem Soc* 110:1657-1666.
3. Jorgensen WL, Chandrasekhar J, Madura R, Impey W, & Klein ML (1983) Comparison of simple potential functions for simulating liquid water. *J Chem Phys* 79:926-35.
4. Kaiser HJ, *et al*. (2011) Lateral sorting in model membranes by cholesterol-mediated hydrophobic matching. *Proc Natl Acad Sci USA* 108(40):16628-16633.
5. Stepniewski M, Bunker A, Pasenkiewicz-Gierula M, Karttunen M, & Rog T (2010) Effects of the lipid bilayer phase state on the water membrane interface. *J Phys Chem B* 114(36):11784-11792.
6. Hess B, Bekker H, Berendsen HJC, & Fraaije JGEM (1997) LINCS: A linear constraint solver for molecular simulations. *J Comput Chem* 18(12):1463-1472.
7. Parinello M & Rahman A (1981) Polymorphic transitions in single crystals: A new molecular dynamics method. *J Appl Phys* 52:7182-90.
8. Bussi G, Donadio D, & Parinello M (2007) Canonical sampling through velocity rescaling. *J Chem Phys* 126:14101-14107.
9. Essmann U, *et al*. (1995) A smooth particle mesh Ewald method. *J Chem Phys* 103(19):8577-8593.
10. Becke AD (1993) Density‐functional thermochemistry. III. The role of exact exchange. *J Chem Phys* 98(7):5648-5652.
11. Frisch MJ, *et al*. (2009) Gaussian 09 (Gaussian, Inc., Wallingford, CT, USA).
12. Breneman CM & Wiberg KB (1990) Determining atom-centered monopoles from molecular electrostatic potentials. The need for high sampling density in formamide conformational analysis. *J Comput Chem* 11(3):361-373.
13. De Proft F, Martin JML, & Geerlings P (1996) On the performance of density functional methods for describing atomic populations, dipole moments and infrared intensities. *Chem Phys Lett* 250(3):393-401.
14. Lee B & Richards FM (1971) The interpretation of protein structures: estimation of static accessibility. *J Mol Biol* 55:379-400.
15. Kolondra A, *et al*. (2010) The role of hydrophobic interactions in ankyrin-spectrin complex formation. *Biochim Biophys Acta* 1798(11):2084-2089.
